# Robust HMM-Based Remaining Useful Life Estimation Using a Ridge-Regularized EM Algorithm

**DOI:** 10.3390/s26041321

**Published:** 2026-02-18

**Authors:** Halime Beyza Küçükdağ, Gokhan Kirkil, Mustafa Hekimoğlu

**Affiliations:** 1 Department of Computational Applied Science and Engineering, Kadir Has University, Istanbul 34083, Turkey; gokhan.kirkil@khas.edu.tr; 2 Department of Industrial Engineering, Faculty of Engineering, MEF University, Istanbul 34396, Turkey; hekimoglum@mef.edu.tr

**Keywords:** remaining useful life, hidden Markov models, ridge regression, EM algorithm, robust statistics, Huber loss, condition monitoring

## Abstract

Estimating the remaining useful life (RUL) of engineering systems is crucial for maintenance planning and the reliability of complex mechanical units. Accurate RUL predictions support timely interventions and help to prevent unexpected failures. This study proposes a statistically robust framework that models degradation signals up to the end of life using a hidden Markov model (HMM) with a simple-failure structure and an absorbing terminal state. The proposed method estimates state-dependent linear emission parameters and transition probabilities using a ridge-regularized expectation–maximization (EM) algorithm. The ridge penalty stabilizes slope estimates under limited data, while a robust Huber-based scale estimator reduces sensitivity to outliers in the sensor-derived health indicator. RUL is computed as a weighted expected time to absorption, combining transient-state survival characteristics with smoothed posterior-state probabilities obtained via the forward–backward algorithm. This yields a low-variance state-aware estimator that preserves the probabilistic structure of the HMM. Simulation studies show that the proposed ridge-regularized EM significantly reduces parameter variance and improves predictive accuracy compared with the baseline weighted least squares EM (WLS-EM). A real-data case analysis demonstrates further improvements in RUL estimation accuracy and smoother, more reliable prediction trajectories. Overall, the framework provides a robust and interpretable approach for practical prognostics applications.

## 1. Introduction

Predicting the remaining useful life (RUL) of engineering systems is a central problem in prognostics and health management (PHM), with direct implications for condition-based maintenance, safety, and operational efficiency. In recent years, both data-driven and model-based approaches have been developed to exploit condition-monitoring (CM) signals collected from sensors embedded in industrial assets. Classical stochastic degradation models, including Wiener-process- and Gamma-process-based formulations [1], have been successfully applied to soft-failure scenarios, where failure is defined by a known degradation threshold. However, such models typically rely on smooth and monotonic degradation trajectories and may be inadequate for systems exhibiting abrupt failures, heterogeneous degradation rates, or latent failure mechanisms. These limitations have motivated increasing interest in hard-failure prognostics, where the relationship between observed signals and failure time is indirect or unobservable [2].

In many practical PHM applications, high-dimensional CM data are first compressed into a univariate health indicator (HI) through feature extraction or dimensionality reduction, after which RUL prediction is performed on the resulting trajectory. Traditional machine learning approaches operating on such health indicators often rely on extensive feature engineering, which requires substantial expert knowledge and manual design effort, making the modeling process costly and difficult to scale across heterogeneous systems [3]. These limitations have motivated increasing interest in end-to-end data-driven models that operate directly on raw sensor sequences. However, irrespective of whether features are manually engineered or automatically learned, RUL prediction methods that rely on explicit failure thresholds become difficult to justify when such thresholds are unknown or system-dependent. State-based degradation models provide a principled alternative by representing the degradation process through discrete latent health states that evolve stochastically over time [4]. Hidden Markov models (HMMs) are particularly attractive in this setting as they decouple latent degradation dynamics from noisy observations and enable scalable inference via efficient forward–backward recursions [5,6,7].

The HMM-based joint modeling framework proposed by Deep et al. [8] demonstrated that integrating CM signals with failure-event information through an absorbing-state structure enables threshold-free RUL estimation as failure is defined by entry into a latent absorbing state rather than by crossing an explicit degradation threshold. By modeling failure as an absorbing terminal state, the expected remaining lifetime can be computed analytically using state posterior probabilities and state-dependent survival characteristics. This formulation has established HMMs as a powerful tool for hard-failure prognostics and has inspired a growing body of follow-up work.

Despite this appeal, recent studies have increasingly emphasized that the practical reliability of HMM-based RUL predictors is often limited not by model structure but estimation stability [8,9]. Related challenges have also motivated Bayesian filtering approaches for RUL prediction, including Kalman and particle filtering methods, which provide a principled framework for sequential state estimation and uncertainty quantification [10]. Nevertheless, their practical performance can be sensitive to prior specification, noise model assumptions, and computational burden, particularly under non-Gaussian disturbances and limited run-to-failure data. First, parameter estimation via maximum likelihood or expectation–maximization (EM) can suffer from severe variance inflation when the effective sample size assigned to individual latent states—implicitly inferred through posterior-state probabilities—is small, a common situation in run-to-failure datasets with short trajectories or unbalanced state occupancy [11,12]. Second, CM-derived health indicators frequently exhibit outliers, transient spikes, and local irregularities arising from operating condition variability or sensor drift, which violate Gaussian noise assumptions and can destabilize parameter estimates [13,14,15]. Third, under a limited number of run-to-failure units, reliance on a single train–test partition without additional variance-control mechanisms may lead to high-variance performance estimates, making model comparison sensitive to the chosen dataset split [9].

Recent advances in robust and regularized learning provide potentially important insights into addressing these challenges, particularly in contrast to physics-based prognostic models. While physics-based approaches can offer strong interpretability, their practical performance is often highly sensitive to the availability and precision of domain knowledge, which may be incomplete or unreliable under complex operating conditions and noisy environments [16]. Moreover, many physics-based models lack the flexibility to be updated online using streaming sensor data, limiting their robustness and adaptability in real-world PHM applications. In this context, robust estimation techniques based on Huber’s seminal work [13,14] have been shown to substantially reduce the influence of anomalous observations and transient outliers in time-series regression and state-space models, providing a principled mechanism to improve estimation stability under non-Gaussian noise. In parallel, regularized EM formulations for latent-variable models have gained attention as a means of controlling estimator variance under limited sample sizes. In particular, ridge-regularized expectation–maximization formulations have been shown to improve numerical conditioning and variance control in regression-based parameter updates for latent-variable models [12]. In parallel, recent studies integrating robust loss principles with latent-state inference highlight the importance of controlling estimator variance and mitigating the influence of anomalous observations in sequential decision-making problems [15,17]. However, the existing approaches typically address these issues in isolation. Regularization-based methods focus on stabilizing regression estimates under data scarcity, while robust estimation techniques primarily target resilience to outliers in the observation model. In HMM-based prognostics trained via EM, these two sources of instability interact non-trivially through posterior-state assignments so that addressing only one of them is often insufficient. This motivates a unified treatment that jointly stabilizes state-dependent slope estimation and residual variance updates within the EM loop.

Motivated by these developments, this paper focuses on stabilizing the estimation of emission parameters in HMM-based hard-failure prognostics. Rather than modifying the latent-state inference mechanism, we target the dominant source of estimation variability: the state-wise regression models used during the parameter update stage of the EM algorithm. Specifically, we develop a ridge-regularized EM framework in which only slope parameters are penalized, a standard practice in regularized regression to preserve interpretable intercepts while controlling variance inflation due to collinearity or limited effective sample size [18,19]. Regularization strengths are selected via cross-validation to avoid ad hoc tuning [20]. In addition, we incorporate a Huber-based robust scale estimator for residual variance estimation, mitigating the impact of outliers and transient anomalies in the health indicator without altering the underlying EM optimization structure.

The resulting model is formulated within a simple-failure HMM structure, where failure is represented as an absorbing state. RUL estimation is performed analytically as the posterior-weighted expected hitting time of the absorbing state using smoothed posterior-state probabilities and transient-state survival characteristics, yielding a low-variance and computationally efficient predictor. Importantly, the proposed approach preserves the interpretability and computational advantages of classical HMM-based joint modeling while explicitly addressing bias–variance trade-offs and robustness at the parameter-estimation level.

The proposed methodology is evaluated through extensive Monte Carlo simulations and a real-world case study. The results demonstrate that the proposed ridge-regularized and robust EM approach consistently improves parameter stability and RUL prediction accuracy relative to the baseline weighted least squares EM (WLS-EM) method, particularly under limited sample sizes and noisy observation regimes.

The main contributions of this work are summarized as follows:

- We develop a ridge-regularized EM algorithm for HMM-based degradation modeling, employing slope-only penalization with cross-validated regularization strengths to stabilize posterior-weighted emission regressions for latent states under data scarcity.
- We incorporate a Huber-based robust residual variance estimator into the M-step, enhancing resistance to outliers and local irregularities in sensor-derived health indicators.
- We retain an analytically tractable state-based RUL estimator within a HMM framework, enabling efficient and low-variance remaining-life prediction.
- We provide comprehensive empirical validation through simulation and empirical data, demonstrating consistent improvements over classical WLS-EM-based approaches.

The remainder of the paper is organized as follows. Section 2 introduces the proposed modeling framework, including the health indicator construction, the HMM specification, and the regularized and robust parameter estimation procedure. Section 3 presents a simulation study under varying sample sizes to assess estimation stability and predictive performance. Section 4 and  Section 5 report the benchmark results on the real dataset. Section 6 discusses the statistical implications, robustness and practical significance of the proposed approach. Finally, Section 7 concludes the paper.

## 2. Materials and Methods

This section presents the complete methodological pipeline in three major components: The proposed robust hidden Markov model (HMM) framework, including the model formulation, EM estimation, ridge–WLS updates, robust scale estimation, and the weighted RUL calculation.

### 2.1. Mathematical Model

We begin by formalizing the statistical structure of the degradation and failure process within a hidden Markov model (HMM) framework. The HMM formulation provides a natural representation for systems whose degradation evolves through latent health states and is observed indirectly through noisy condition-monitoring (CM) signals. This perspective is consistent with state-based prognostic models used in PHM applications [8] and is rooted in classical probabilistic modeling introduced by Baum and colleagues in the 1960s [13] as extensions of Markov’s early work on sequential dependence.

#### 2.1.1. Degradation and Failure Behavior

Consider a system with *N* identical units operating under similar conditions and subject to only corrective maintenance (run-to-failure). For each unit i∈{1,…,N}, a monitoring system collects degradation indicators periodically. Let $y_{it}$ denote a scalar degradation indicator observed at period $t=1,\ldots ,T_{i}$. Such indicators are assumed to summarize the dominant degradation dynamics from multivariate condition-monitoring signals and are treated as observed inputs to the prognostic model. The observation horizon $T_{i}$ varies across units, and each trajectory terminates upon failure. While failure time $T_{i}$ is observed, the underlying degradation level that triggers failure is latent, and it is necessary to infer degradation stages from $y_{it}$ and failure times [21].

#### 2.1.2. Hidden Markov Model Structure

The latent degradation process is modeled as a discrete-time hidden Markov model (HMM) with finite state space S={1,…,K}. Let $z_{it}$ denote the latent degradation state of unit *i* at time *t*. States 1 to K−1 correspond to progressive degradation levels, while the state *K* represents the failure point, which is modeled as an absorbing state. All units are assumed to start at the initial healthy state, $z_{i1}=1$.

The state dynamics follow a first-order Markov chain with transition matrix $P=[p_{k\ell }]$, where

$$P\left(z_{t+1}=\ell |z_{t}=k\right)=p_{k\ell },\;k,\ell \in \left\{1,\ldots ,K\right\}.$$

The failure state *K* is absorbing, i.e., $p_{KK}=1$, $p_{K\ell }=0$ for ℓ<K, and $p_{kK}>0$ for k<K. The failure time, denoted by τ, is defined as the first hitting time to the absorbing state, starting from state 1 at time 1:

$$\tau =\inf\{t\geq 1:z_{t}=K\}.$$

The relationship between the transition matrix of *P* and absorption-time quantities underlying remaining useful life (RUL) estimation is classical.

#### 2.1.3. State-Dependent Emission Model

Conditioned on the latent state $z_{t}$, the degradation signal $y_{t}$ follows a Gaussian emission distribution with a state-specific linear mean structure. For each state k∈{1,…,K} and time t∈{1,…,T},

(1) $$y_{t}=\eta_{0,k}+\eta_{1,k}\;t+\varepsilon_{t,k},\;\varepsilon_{t,k}∼N\left(0,\sigma_{k}^{2}\right),\;\mathrm{when}\;z_{t}=k,$$

where $\eta_{0,k}$ and $\eta_{1,k}$ denote the state-dependent intercept and slope, respectively, and $\sigma_{k}^{2}$ is the corresponding noise variance.

This state-wise linear formulation captures gradual but potentially heterogeneous degradation behavior across latent states. Following the HMM-based joint modeling framework for condition-monitoring signals and failure events [8], we approximate the degradation dynamics within each latent health state by a simple linear trend, which provides a parsimonious yet interpretable representation of state-dependent degradation behavior.

The emission probability density associated with state *k* is therefore given by

(2) $$b_{k}\left(y_{t}\right)=N\;\left(y_{t}\;;\;\eta_{0,k}+\eta_{1,k}\;t,\;\sigma_{k}^{2}\right),$$

and the emission parameters for each state are collected as

$$\theta_{k}=\left(\eta_{0,k},\;\eta_{1,k},\;\sigma_{k}\right).$$

Under the standard conditional independence assumption of hidden Markov models, the joint likelihood of the observation sequence given the latent-state path factorizes as

(3) $$p\left(y_{1:T}|z_{1:T}\right)=\prod_{t=1}^{T}b_{z_{t}}\left(y_{t}\right)$$

where $b_{k}\left(\cdot \right)$ denotes the Gaussian emission density associated with state *k*.

This emission model integrates naturally into the Baum–Welch EM framework, where posterior-state probabilities obtained from the forward–backward algorithm are used to perform weighted estimation of the state-dependent parameters. This formulation provides the foundation for the regularized and robust emission updates introduced in the subsequent sections.

### 2.2. Complete Likelihood Representation

Under the hidden Markov model formulation, the joint distribution of the latent state sequence $z_{1:T}$ and the observed degradation signal $y_{1:T}$ admits a standard factorization into transition and emission components. This structure enables parameter estimation via the expectation–maximization (EM) algorithm, where latent states are treated as missing data [22].

For a single unit with latent states $z_{t}\in \left\{1,\ldots ,K\right\}$ and observations $y_{1:T}=\left(y_{1},\ldots ,y_{T}\right)$, the complete-data likelihood is given by

(4) $$L\left(y_{1:T},z_{1:T}|\Theta \right)=P\left(z_{1}\right)\prod_{t=1}^{T-1}p_{z_{t}z_{t+1}}\prod_{t=1}^{T}b_{z_{t}}\left(y_{t}\right),$$

where $P(z_{1})$ denotes the initial state distribution, the transition probabilities $p_{k\ell }$ are defined in Section 2.1.2. This likelihood corresponds to the classical complete-data formulation for HMMs [5,6].

Failure information in the present work is encoded via an absorbing-state HMM, which preserves the standard likelihood while enabling RUL estimation through absorption-time properties of the transition matrix. While transition probabilities admit stable closed-form updates, estimation of the linear emission parameters may become ill-conditioned when only a limited number of observations effectively contribute to a given latent state. We therefore adopt a ridge-regularized weighted least squares surrogate in the M-step (Section 2.3.3).

### 2.3. Robust HMM Framework for Hard-Failure Prognostics

Our proposed framework builds upon the standard absorbing-state HMM formulation. Rather than reiterating the classical HMM and EM machinery, we focus on robustness under limited and noisy degradation data.

With the state-space formulation specified in Section 2, the degradation process is fully characterized by (i) a first-order Markov transition model with an absorbing failure state and (ii) a state-dependent Gaussian emission model. Together, these components define a complete-data likelihood that admits a natural decomposition into transition and emission terms. This decomposition is central to likelihood-based inference for HMMs and leads directly to an expectation–maximization (EM) estimation scheme. We therefore proceed by deriving the EM updates, beginning with the computation of the smoothed posterior-state probabilities via the forward–backward (Baum–Welch) recursion.

#### 2.3.1. E-Step: Forward–Backward Smoothing

For each unit i∈{1,…,N}, let $y_{it}$ denote the degradation signal observed at time $t=1,\ldots ,T_{i}$, and let $z_{it}\in \left\{1,\ldots ,K\right\}$ denote the corresponding latent degradation state. The initial state distribution is denoted by $\pi =(\pi_{1},\ldots ,\pi_{K})$, where

$$\pi_{k}=P\left(z_{i1}=k\right),\;k=1,\ldots ,K,$$

and, in the hard-failure setting considered here, all units are assumed to start in the healthy initial state,

$$\pi_{1}=1,\;\pi_{k}=0\;\mathrm{for}\;k>1.$$

The E-step evaluates the conditional expectations of latent-state indicators given the current parameter estimates. This is carried out using the classical forward–backward recursion (Baum–Welch algorithm) [5,6], summarized below.

**Forward variables.** For unit *i*, the forward variables

$$\alpha_{it}\left(k\right)=P\left(y_{i,1:t},\;z_{it}=k|\Theta \right),\;t=1,\ldots ,T_{i},\;\;k=1,\ldots ,K,$$

are computed recursively as

(5) $$\alpha_{i1}\left(k\right)=\pi_{k}\;b_{k}\left(y_{i1}\right),\;k=1,\ldots ,K,$$

(6) $$\alpha_{it}\left(k\right)=b_{k}\left(y_{it}\right)\sum_{\ell =1}^{K}\alpha_{i,t-1}\left(\ell \right)\;p_{\ell k},\;t=2,\ldots ,T_{i},\;\;k=1,\ldots ,K,$$

where $b_{k}\left(\cdot \right)$ denotes the Gaussian emission density associated with latent state *k*, as defined in (2). The summation term aggregates the probability of transitioning into state *k* at time *t* from all possible latent states at time t−1, while the factor $b_{k}\left(y_{it}\right)$ accounts for the likelihood of observing $y_{it}$ in state *k*.

**Backward variables.** The backward variables

$$\beta_{it}\left(k\right)=P\left(y_{i,t+1:T_{i}}|z_{it}=k,\Theta \right),\;t=1,\ldots ,T_{i},\;\;k=1,\ldots ,K,$$

are initialized at the final time and propagated backwards as

(7) $$\beta_{i,T_{i}}\left(k\right)=1,\;k=1,\ldots ,K,$$

(8) $$\beta_{it}\left(k\right)=\sum_{\ell =1}^{K}p_{k\ell }\;b_{\ell }\left(y_{i,t+1}\right)\;\beta_{i,t+1}\left(\ell \right),\;t=T_{i}-1,\ldots ,1,\;\;k=1,\ldots ,K.$$

**Posterior-state probabilities (smoothing).** For each unit *i*, the posterior probability of being in state *k* at time *t* is

(9) $$\gamma_{it}\left(k\right)=P\left(z_{it}=k|y_{i,1:T_{i}},\Theta \right)=\frac{\alpha_{it}\left(k\right)\;\beta_{it}\left(k\right)}{\sum_{m=1}^{K}\alpha_{it}\left(m\right)\;\beta_{it}\left(m\right)},\;t=1,\ldots ,T_{i},\;\;k=1,\ldots ,K.$$

The denominator corresponds to the marginal likelihood $L_{i}=p\left(y_{i,1:T_{i}}|\Theta \right)$ and does not depend on *t*. Consequently, $\gamma_{it}\left(k\right)=\alpha_{it}\left(k\right)\beta_{it}\left(k\right)/L_{i}$ for all *t*.

**Joint posterior of successive states.** For each unit *i*, the joint posterior probability of being in state *k* at time *t* and state *ℓ* at time t+1 is

(10) $$\xi_{it}\left(k,\ell \right)=P\left(z_{it}=k,\;z_{i,t+1}=\ell |y_{i,1:T_{i}},\Theta \right),\;t=1,\ldots ,T_{i}-1,\;\;k,\ell =1,\ldots ,K.$$

Using the forward–backward variables, this quantity can be expressed as

(11) $$\xi_{it}\left(k,\ell \right)=\frac{\alpha_{it}\left(k\right)\;p_{k\ell }\;b_{\ell }\left(y_{i,t+1}\right)\;\beta_{i,t+1}\left(\ell \right)}{L_{i}}.$$

The smoothed probabilities $\gamma_{it}\left(k\right)$ quantify the unit- and time-specific responsibility of latent state *k* for each observation and are used as weights in the update of the emission regression parameters. The joint probabilities $\xi_{it}\left(k,\ell \right)$ aggregate expected transition counts and yield closed-form updates of the transition probability matrix in the parameter estimation step.

#### 2.3.2. M-Step: Transition Matrix and Weighted Least Squares (WLS) Emission Update

Given the smoothed state posteriors $\gamma_{it}\left(k\right)$ and joint transition posteriors $\xi_{it}\left(k,\ell \right)$ obtained from the E-step, the parameter estimation step updates the transition probabilities by maximizing the expected complete-data log-likelihood. For a first-order Markov chain, this leads to a closed-form estimator in which each row of the transition matrix corresponds to normalized expected transition counts. This result is standard in EM estimation for hidden Markov models and follows directly from the structure of the complete-data log-likelihood (see, e.g., [5,6,23]).

(12) $${\hat{p}}_{k\ell }=\frac{\sum_{i=1}^{N}\sum_{t=1}^{T_{i}-1}\xi_{it}\left(k,\ell \right)}{\sum_{i=1}^{N}\sum_{t=1}^{T_{i}-1}\gamma_{it}\left(k\right)},\;k,\ell =1,\ldots ,K.$$

The absorbing-state constraints in () are enforced after each row update to ensure that state *K* remains absorbing under the simple-failure structure.

As a reference (unregularized) estimator, emission parameters are updated using a standard weighted least squares (WLS) procedure within the EM framework. Conditioned on the smoothed state posteriors $\gamma_{it}\left(k\right)$ obtained from the E-step and the linear Gaussian emission model defined in (1), the state-wise WLS update for each transient state k∈{1,…,K−1} is obtained by solving

(13) $$\left({\hat{\eta }}_{0k},{\hat{\eta }}_{1k}\right)=\arg\min_{\eta_{0},\eta_{1}}\sum_{i=1}^{N}\sum_{t=1}^{T_{i}}\gamma_{it}\left(k\right)\;{\left(y_{it}-\eta_{0}-\eta_{1}t\right)}^{2}.$$

Given the resulting fitted values ${\hat{y}}_{it,k}$, the state-specific noise variance is updated by

(14) $${\hat{\sigma }}_{k}^{2}=\frac{\sum_{i,t}\gamma_{it}\left(k\right)\;{\left(y_{it}-{\hat{y}}_{it,k}\right)}^{2}}{\sum_{i,t}\gamma_{it}\left(k\right)}.$$

The closed-form expressions for the WLS estimators follow directly from the normal equations and are standard in HMM-based regression models (e.g., [5,6,8]). In the proposed framework, the WLS-EM formulation is used solely as a baseline to assess the benefits of the ridge-regularized and robust emission updates introduced in the next subsection. For completeness, the explicit closed-form solutions are reported in Appendix A.

#### 2.3.3. M-Step: Ridge-Regularized Emission Update

To improve numerical stability of the emission parameter estimates under limited effective state-specific sample support, we replace the unregularized WLS update with a ridge-regularized weighted least squares formulation. Here, the effective sample size for each state is induced by the posterior-state probabilities obtained in the E-step rather than by directly observed state-labeled data. The regularization is applied only to the slope parameter in order to stabilize the estimated degradation rate while preserving the interpretability of the state-dependent intercept.

For each transient state k∈{1,…,K−1}, the emission parameters $\left(\eta_{0k},\eta_{1k}\right)$ are obtained by minimizing the penalized objective

(15) $$\left({\hat{\eta }}_{0k},{\hat{\eta }}_{1k}\right)=\arg\min_{\eta_{0},\eta_{1}}\sum_{i=1}^{N}\sum_{t=1}^{T_{i}}\gamma_{it}\left(k\right)\;{\left(y_{it}-\eta_{0}-\eta_{1}t\right)}^{2}+\frac{1}{2}\;\lambda_{k}\;\eta_{1}^{2},$$

where $\lambda_{k}\geq 0$ is a state-specific regularization parameter. The resulting penalized objective coincides with the classical ridge regression criterion, which augments the weighted least squares loss with a quadratic $\ell_{2}$ penalty on the regression coefficients to improve numerical stability [18]. The factor 1/2 is a matter of convention and simplifies derivatives.

Let $n_{k}$ denote the effective number of observations associated with state *k*. Define the design matrix $X\in R^{n_{k}\times 2}$ with rows (1,t), the observation vector $y\in R^{n_{k}}$ collecting the corresponding measurements $y_{it}$, and the diagonal weight matrix

$$W_{k}=\mathrm{diag}\left(\gamma_{i_{1}t_{1}}\left(k\right),\ldots ,\gamma_{i_{n_{k}}t_{n_{k}}}\left(k\right)\right),$$

which aggregates the posterior-state probabilities obtained from the E-step. Under this notation, (15) can be written compactly as

(16) $${\hat{\theta }}_{k}=\arg\min_{\theta \in R^{2}}{\left(y-X\theta \right)}^{⊤}W_{k}\left(y-X\theta \right)+\frac{1}{2}\;\lambda_{k}\;\theta^{⊤}D\;\theta ,$$

where $\theta ={\left(\eta_{0k},\eta_{1k}\right)}^{⊤}$ and D=diag(0,1) enforces slope-only penalization. This formulation shows explicitly that the emission update corresponds to a posterior-weighted ridge regression problem, in which uncertainty about latent-state assignments is accounted for through $W_{k}$.

The resulting estimator is obtained from the modified normal equations

(17) $${\hat{\theta }}_{k}^{\mathrm{Ridge}}={\left(X^{⊤}W_{k}X+\frac{1}{2}\lambda_{k}D\right)}^{-1}X^{⊤}W_{k}y,\;{\hat{\theta }}_{k}^{\mathrm{Ridge}}={\left({\hat{\eta }}_{0k},{\hat{\eta }}_{1k}\right)}^{⊤}.$$

In implementation, these quantities are computed from weighted sufficient statistics rather than explicit construction of *X* and $W_{k}$, improving both numerical stability and computational efficiency.

#### 2.3.4. Selection of the Regularization Strength via Cross-Validation

For each latent state *k*, the ridge regularization parameter $\lambda_{k}$ is selected in a data-driven manner using *K*-fold cross-validation, which is performed exclusively within the training set to select the ridge penalty, while all reported performance metrics are computed on a held-out test set. The optimal amount of regularization depends on the effective sample size and noise level within each state and therefore cannot be fixed a priori.

**State-specific weighted observation set.** The state-specific weighted observation set associated with state *k* is defined as

(18) $$D_{k}=\left\{\left(i,t\right):\gamma_{it}\left(k\right)>0\right\},$$

where each observation (i,t) is weighted by its posterior responsibility $\gamma_{it}\left(k\right)$.

***K*****-fold partitioning.** The index set $D_{k}$ is randomly partitioned into *K* disjoint folds,

(19) $$D_{k}=\overset{K}{\underset{j=1}{⋃}}D_{k}^{\left(j\right)},\;D_{k}^{\left(j\right)}\cap D_{k}^{\left(\ell \right)}=⌀\;\mathrm{for}\;j\neq \ell .$$

**Training loss.** For a given candidate value λ∈Λ and fold *j*, the ridge-regularized weighted least squares estimator is obtained by minimizing the training objective

(20) $$\left({\hat{\eta }}_{0k}^{\left(j\right)}\left(\lambda \right),{\hat{\eta }}_{1k}^{\left(j\right)}\left(\lambda \right)\right)=\arg\min_{\eta_{0},\eta_{1}}\sum_{\left(i,t\right)\in D_{k}\setminus D_{k}^{\left(j\right)}}\gamma_{it}\left(k\right)\;{\left(y_{it}-\eta_{0}-\eta_{1}t\right)}^{2}+\frac{\lambda }{2}\;\eta_{1}^{2},$$

where the ridge penalty is applied only during training.

**Validation loss.** The corresponding validation loss on the held-out fold $D_{k}^{\left(j\right)}$ is defined as

(21) $$\ell_{k}^{\left(j\right)}\left(\lambda \right)=\sum_{\left(i,t\right)\in D_{k}^{\left(j\right)}}\gamma_{it}\left(k\right)\;{\left(y_{it}-{\hat{\eta }}_{0k}^{\left(j\right)}\left(\lambda \right)-{\hat{\eta }}_{1k}^{\left(j\right)}\left(\lambda \right)t\right)}^{2},$$

which does not include any regularization term.

**Cross-validation score.** Averaging the validation loss across folds yields the cross-validation score

(22) $${\mathrm{CV}}_{k}\left(\lambda \right)=\frac{1}{K}\sum_{j=1}^{K}\ell_{k}^{\left(j\right)}\left(\lambda \right).$$

**Standard error estimation.** The variability of the validation loss across folds is quantified by

(23) $${\mathrm{SD}}_{k}\left(\lambda \right)=\sqrt{\frac{1}{K-1}\sum_{j=1}^{K}{\left(\ell_{k}^{\left(j\right)}\left(\lambda \right)-{\mathrm{CV}}_{k}\left(\lambda \right)\right)}^{2}},$$

(24) $${\mathrm{SE}}_{k}\left(\lambda \right)=\frac{{\mathrm{SD}}_{k}\left(\lambda \right)}{\sqrt{K}},$$

where ${\mathrm{SE}}_{k}\left(\lambda \right)$ measures the uncertainty of the estimated mean validation loss.

**One-standard-error (1 − SE) rule.** Let ${\mathrm{CV}}_{k}\left(\lambda \right)$ denote the mean K-fold cross-validation loss (weighted mean squared error) computed over the state-specific weighted observation set $D_{k}$

(25) $$\lambda_{\min}=\arg\min_{\lambda \in \Lambda }{\mathrm{CV}}_{k}\left(\lambda \right)$$

denote the value minimizing the cross-validation score. The admissible set of regularization parameters is defined as

(26) $$A_{k}=\left\{\lambda \in \Lambda :{\mathrm{CV}}_{k}\left(\lambda \right)\leq {\mathrm{CV}}_{k}\left(\lambda_{\min}\right)+{\mathrm{SE}}_{k}\left(\lambda_{\min}\right)\right\}.$$

Finally, we select

(27) $$\lambda_{k}=\max A_{k},$$

which favors the strongest regularization that remains statistically indistinguishable from the minimum validation error. This choice yields a more stable estimator and mitigates overfitting under limited effective sample sizes [18].

Once the state-specific regularization strengths $\lambda_{k}$ have been selected and the ridge-regularized emission parameters $\left({\hat{\eta }}_{0k},{\hat{\eta }}_{1k}\right)$ are obtained, the remaining component of the M-step concerns the estimation of the state-dependent noise variance.

#### 2.3.5. Robust Variance Estimation

Given the ridge-regularized emission estimates $\left({\hat{\eta }}_{0k},{\hat{\eta }}_{1k}\right)$, we update the state-specific noise variance using a robust M-estimator of scale in order to limit the influence of atypical residuals and local deviations from the assumed Gaussian noise model.

Specifically, we define the fitted values $\left({\hat{\eta }}_{0k},{\hat{\eta }}_{1k}\right)$ as

(28) $${\hat{y}}_{it,k}={\hat{\eta }}_{0k}+{\hat{\eta }}_{1k}\;t,$$

and update the state-specific noise variance using a Huber-type robust M-estimator of scale [13,14,24]. Let $r_{it,k}=y_{it}-{\hat{y}}_{it,k}$ denote the residuals, and let ${\mathrm{MAD}}_{k}$ be the weighted median absolute deviation of the set $\left\{r_{it,k}\right\}$ with weights $\gamma_{it}\left(k\right)$. An initial robust scale is obtained through the standard normal consistency factor 0.6745, i.e.,

$${\hat{s}}_{0k}=\frac{{\mathrm{MAD}}_{k}}{0.6745}.$$

To achieve 95% asymptotic efficiency under Gaussian noise, we adopt the classical Huber threshold of 1.345 [13]. We therefore set

$$\delta_{k}=1.345\;\max\;\left({\hat{s}}_{0k},\;\varepsilon \right),$$

with a small ε>0 to prevent degeneracy. The Huber loss function is

$$\rho_{\delta_{k}}\left(r\right)=\left\{\begin{array}{cc} r^{2}, & \left|r\right|\leq \delta_{k},\\ 2\delta_{k}\left|r\right|-\delta_{k}^{2}, & |r|>\delta_{k}, \end{array}\right.$$

which yields a bounded-influence scale update. The state-wise noise variance is estimated by

(29) $${\hat{\sigma }}_{k}^{2}=\frac{\sum_{i=1}^{N}\sum_{t=1}^{T_{i}}\gamma_{it}\left(k\right)\;\rho_{\delta_{k}}\left(r_{it,k}\right)}{\sum_{i=1}^{N}\sum_{t=1}^{T_{i}}\gamma_{it}\left(k\right)}.$$

This robust variance update reduces the influence of occasional large residuals that can disproportionately affect the standard WLS variance estimator, particularly in the presence of local anomalies or short-lived deviations from the assumed linear degradation trend. When combined with ridge-regularized slope estimation, the resulting M-step achieves a more favorable bias–variance trade-off by stabilizing both the mean and variance updates without sacrificing efficiency for nominal Gaussian noise [18].

The practical impact of this robustification is illustrated in the simulation study (Section 3), where residual-wise contributions to the variance update under the standard WLS and Huber losses are contrasted.

The complete estimation procedure, integrating the E-step with the ridge-regularized and Huber-robust M-step updates, is summarized in Algorithm 1. The algorithm preserves the classical EM structure while incorporating penalized and robust components in a closed-form and computationally efficient manner. This formulation ensures stable parameter updates across iterations and provides the foundation for the analytical RUL estimator derived in the subsequent section.
**Algorithm 1** Penalized EM Algorithm for the Robust Absorbing-State HMM.**Require:** Observed trajectories ${\left\{y_{it}\right\}}_{t=1}^{T_{i}}$ for i=1,…,N; number of states *K* (state *K* absorbing); ridge penalties ${\left\{\lambda_{k}\right\}}_{k=1}^{K-1}$.
  1:Initialize parameters $\Theta^{\left(0\right)}=\left\{P^{\left(0\right)},\left(\eta_{0,1}^{\left(0\right)},\eta_{1,1}^{\left(0\right)},\sigma_{1}^{2(0)}\right),\ldots ,\left(\eta_{0,K}^{\left(0\right)},\eta_{1,K}^{\left(0\right)},\sigma_{K}^{2(0)}\right)\right\}$ (e.g., simple-failure $P^{\left(0\right)}$ and a global linear regression for emissions).  2:**repeat**  3:      **E-step (forward–backward).**  4:      Run the scaled forward–backward recursion to obtain $\gamma_{it}\left(k\right)$ for $t=1,\ldots ,T_{i}$ and $\xi_{it}\left(k,\ell \right)$ for $t=1,\ldots ,T_{i}-1$ (see (6)–(11)).  5:      **M-step: transition matrix.**  6:      Update$${\hat{p}}_{k\ell }=\frac{\sum_{i=1}^{N}\sum_{t=1}^{T_{i}-1}\xi_{it}\left(k,\ell \right)}{\sum_{i=1}^{N}\sum_{t=1}^{T_{i}-1}\gamma_{it}\left(k\right)},\;k,\ell =1,\ldots ,K.$$  7:      **M-step: emission parameters (ridge-regularized).**  8:      Select $\lambda_{k}$ by weighted *K*-fold cross-validation under the current weights $\gamma_{it}\left(k\right)$.  9:      **for** each transient state k=1,…,K−1 **do**10:            Update $\left(\eta_{0,k},\eta_{1,k}\right)$ by slope-only ridge regression:$$\left(\eta_{0,k},\eta_{1,k}\right)=\arg\min_{\eta_{0},\eta_{1}}\sum_{i=1}^{N}\sum_{t=1}^{T_{i}}\gamma_{it}\left(k\right)\;{\left(y_{it}-\eta_{0}-\eta_{1}t\right)}^{2}+\frac{1}{2}\lambda_{k}\eta_{1}^{2}.$$11:            Update $\sigma_{k}^{2}$ using a Huber-type robust scale estimator based on the weighted residuals.12:      **end for**13:**until** convergence of the log-likelihood and parameter updates.


### 2.4. RUL Estimation via Expected Hitting Time

Under the absorbing-state hidden Markov model, system failure is defined as the first hitting time of the absorbing state *K*. For unit *i*, the failure time is given by

(30) $$\tau_{i}=\inf\left\{t\geq 1:z_{it}=K\right\},\;$$

where $z_{it}$ denotes the latent health state at time *t*. The remaining useful life (RUL) at time $t^{*}$ is then defined as the expected remaining time until absorption, $E[\tau_{i}-t^{*}|Y_{i,1:t^{*}}]$.

For a first-order Markov chain with a single absorbing state, classical results for absorbing Markov chains apply. Let $Q\in R^{\left(K-1)\times (K-1\right)}$ denote the transient-state submatrix of the estimated transition matrix *P*, and define the fundamental matrix [25]

(31) $$F={\left(I-Q\right)}^{-1}.$$

If the latent state at time $t^{*}$ were known to be k∈{1,…,K−1}, the expected remaining time to absorption would be

(32) $$E\left[\tau_{i}-t^{*}|z_{it^{*}}=k\right]=\sum_{s=1}^{K-1}F_{ks},\;$$

which corresponds to the expected number of future visits to transient states starting from state *k*.

In practice, the latent state is not observed. Instead, the EM algorithm provides the smoothed posterior probabilities $\gamma_{it^{*}}\left(k\right)=P\left(z_{it^{*}}=k|y_{i,1:T_{i}},\Theta \right)$. Taking the expectation of (32) with respect to these posteriors yields the RUL estimator

(33) $${\hat{\mathrm{RUL}}}_{i}\left(t^{*}\right)=\sum_{k=1}^{K-1}\gamma_{it^{*}}\left(k\right)\sum_{s=1}^{K-1}F_{ks}.\;$$

Equivalently, this expression can be written in compact vector form as

(34) $${\hat{\mathrm{RUL}}}_{i}\left(t^{*}\right)=\gamma_{it^{*}}^{⊤}F\;1_{K-1},\;$$

where $\gamma_{it^{*}}$ collects the posterior probabilities over the transient states and $1_{K-1}$ denotes the all-ones vector. This closed-form computation is efficient and depends only on the estimated transition matrix *P*.

The absorbing-state (simple-failure) structure ensures that *Q* is strictly sub-stochastic and that (I−Q) is invertible, guaranteeing numerical stability of the RUL estimator.

To sum up, in the proposed framework, each EM iteration consists of (i) an E-step computing the forward–backward recursions and the associated smoothed quantities $\gamma_{it}\left(k\right)$ and $\xi_{it}\left(k,\ell \right)$, and (ii) an M-step updating the transition matrix *P* and the state-wise emission parameters $\left(\eta_{0k},\eta_{1k},\sigma_{k}^{2}\right)$. While the standard EM algorithm monitors the ascent of the (unpenalized) log-likelihood

$$\ell \left(\Theta \right)=\sum_{i=1}^{N}\log p\left(y_{i,1:T_{i}}|\Theta \right),$$

the ridge-regularized M-step instead maximizes the penalized surrogate objective

(35) $$\tilde{Q}\left(\Theta ;\Theta^{\mathrm{old}}\right)=Q\left(\Theta ;\Theta^{\mathrm{old}}\right)-\frac{1}{2}\sum_{k=1}^{K-1}\lambda_{k}\;\eta_{1k}^{2},$$

reflecting the slope-only regularization applied to the emission trends. Accordingly, convergence is assessed using both (i) the absolute change in $\tilde{Q}$ and (ii) the maximum change in the emission parameters across iterations. For interpretability and for comparison with the unregularized WLS-EM baseline, we also record the unpenalized log-likelihood trace $\ell^{\left(r\right)}$ at each iteration *r*.

The selection of the ridge penalties $\lambda_{k}$ is carried out during the early phase of the EM procedure via weighted cross-validation on the state-wise prediction loss using the one-standard-error rule [18]. The resulting values are then kept fixed to stabilize the subsequent emission updates.

Because EM algorithms for latent-state models can be sensitive to initialization, we follow the general guidance of Wu [26] and adopt a carefully designed structured initialization for the transition matrix and regression coefficients. This initialization is kept fixed across runs as the imposed monotonicity and absorbing-state constraints provide sufficient numerical stability and ensure full reproducibility of the final estimates.

The complete posterior-weighted RUL estimation procedure is summarized in Algorithm 2. This formulation provides a systematic and computationally straightforward way to obtain remaining useful life predictions from the estimated HMM parameters. The predictive performance of the proposed framework is evaluated next through Monte Carlo experiments under controlled simulation settings.
**Algorithm 2** Posterior-Weighted RUL Estimation.**Require:** Estimated parameters $\hat{\Theta }$; absorbing state *K*; observed degradation trajectory ${\left\{y_{it}\right\}}_{t=1}^{T_{i}}$ for a new unit *i*.
1:**Emission Evaluation:**  Compute Gaussian emission densities $b_{k}\left(y_{it}\right)$ for all $t=1,\ldots ,T_{i}$ and k=1,…,K using the linear emission model in (1).2:**Forward–Backward Recursions:**  Run the scaled forward and backward recursions ((6)–(8)) to obtain $\alpha_{it}\left(k\right)$ and $\beta_{it}\left(k\right)$.  Compute smoothed posteriors $\gamma_{it}\left(k\right)$ via (9).3:**Fundamental Matrix:**  Extract the transient submatrix $Q={\hat{P}}_{1:(K-1),\;1:(K-1)}$.  Compute the fundamental matrix $F={\left(I_{K-1}-Q\right)}^{-1}$.4:**Compute RUL Path:**  For each $t=1,\ldots ,T_{i}$, evaluate$${\hat{\mathrm{RUL}}}_{i}\left(t\right)=\gamma_{it}^{⊤}F1_{K-1}$$
using (34).5:**return** Full trajectory ${\left\{{\hat{\mathrm{RUL}}}_{i}\left(t\right)\right\}}_{t=1}^{T_{i}}$ and the current estimate ${\hat{\mathrm{RUL}}}_{i}\left(T_{i}\right)$.


## 3. Monte Carlo Simulation Study

This section investigates the statistical properties of the proposed robust HMM estimators under controlled synthetic conditions. The goal is to assess (i) parameter stability, (ii) variance reduction under regularization, and (iii) improvements in RUL prediction accuracy relative to the unregularized WLS-EM baseline.

### 3.1. Simulation Setup

Synthetic run-to-failure trajectories were generated from a *K*-state simple-failure HMM with linear Gaussian emissions as defined in (1) using ground-truth parameters chosen to reproduce monotone degradation patterns typical of PHM applications. The latent-state dynamics follow a progressive structure in which units may remain in the same state or transition only to the next degradation level (i.e., from state *k* to k+1), with state *K* modeled as absorbing. The true transition matrix used in the simulations is provided in Table 1, and the emission parameters $\left(\eta_{0,k},\eta_{1,k},\sigma_{k}\right)$ for each state are listed in Table 2.

For each Monte Carlo replication, *N* independent units were simulated until absorption, with N∈{5,10,20,50,100} and B=100 repetitions per setting. To assess robustness, the simulations incorporated two controlled perturbations:

- Effectively heavy-tailed noise—rare large residuals were introduced through occasional high-magnitude deviations in the emission noise, resulting in heavier-than-Gaussian tails without explicitly changing the nominal noise model.
- Heterogeneous degradation rates—unit-to-unit variability in degradation speed was induced by state-dependent emission slopes, causing different progression rates through the latent health states while preserving the same transition matrix.

### 3.2. Estimation and Evaluation Metrics

Model estimation is performed using the EM algorithm described in Section 2, with either weighted least squares (WLS) or ridge-regularized updates for the state-dependent emission parameters.

**Figure 1:**
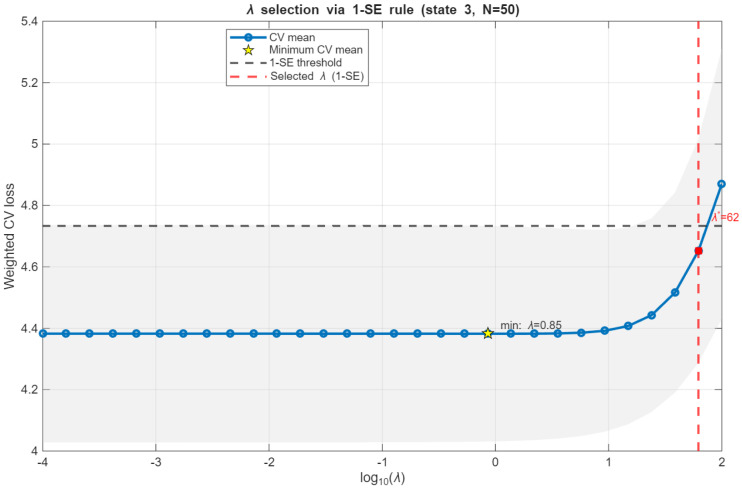
Cross-validation loss curve $CV_{k}\left(\lambda \right)$ as a function of the regularization strength λ for state k=3 with N=50. The blue curve shows the mean *K*-fold validation loss, the star indicates the minimizer $\lambda_{\min}$, and the horizontal dashed line denotes the one-standard-error (1 − SE) threshold. The selected regularization strength $\lambda_{k}$ is marked by the vertical line and corresponds to the largest λ whose validation loss remains within the 1 − SE criterion.

The regularization strengths $\lambda_{k}$ are selected in a data-driven manner via *K*-fold cross-validation on the weighted prediction loss in (15) using the one-standard-error (1 − SE) rule. Candidate values of λ are searched on a fixed logarithmic grid ranging from $10^{-4}$ to $10^{2}$, with 30 evenly spaced points on the logarithmic scale, which is shared between all states to ensure comparability of the regularization path. As illustrated in Figure 1, the cross-validation loss curve $CV_{k}\left(\lambda \right)$ typically exhibits a flat minimum over a wide range of values λ, enabling the use of the 1 − SE rule to select stable regularization. Consequently, for each state *k*, we select the largest λ whose validation loss is statistically indistinguishable from the minimum, producing a conservative state-dependent choice that prioritizes numerical stability under limited effective sample sizes.

The performance of the model in the Monte Carlo experiments is evaluated using complementary metrics that capture both the accuracy of the estimation and the predictive reliability. Specifically, we report: (i) parameter-space mean squared error (MSE) for the emission parameters and transition probabilities; (ii) predictive MSE of the one-step-ahead emission means; and (iii) RUL prediction accuracy, assessed via the RMSE of $\hat{\mathrm{RUL}}\left(t\right)$ over time and across Monte Carlo replications, together with a bias–variance decomposition of the RUL MSE.

### 3.3. Simulation Results

**Figure 2:**
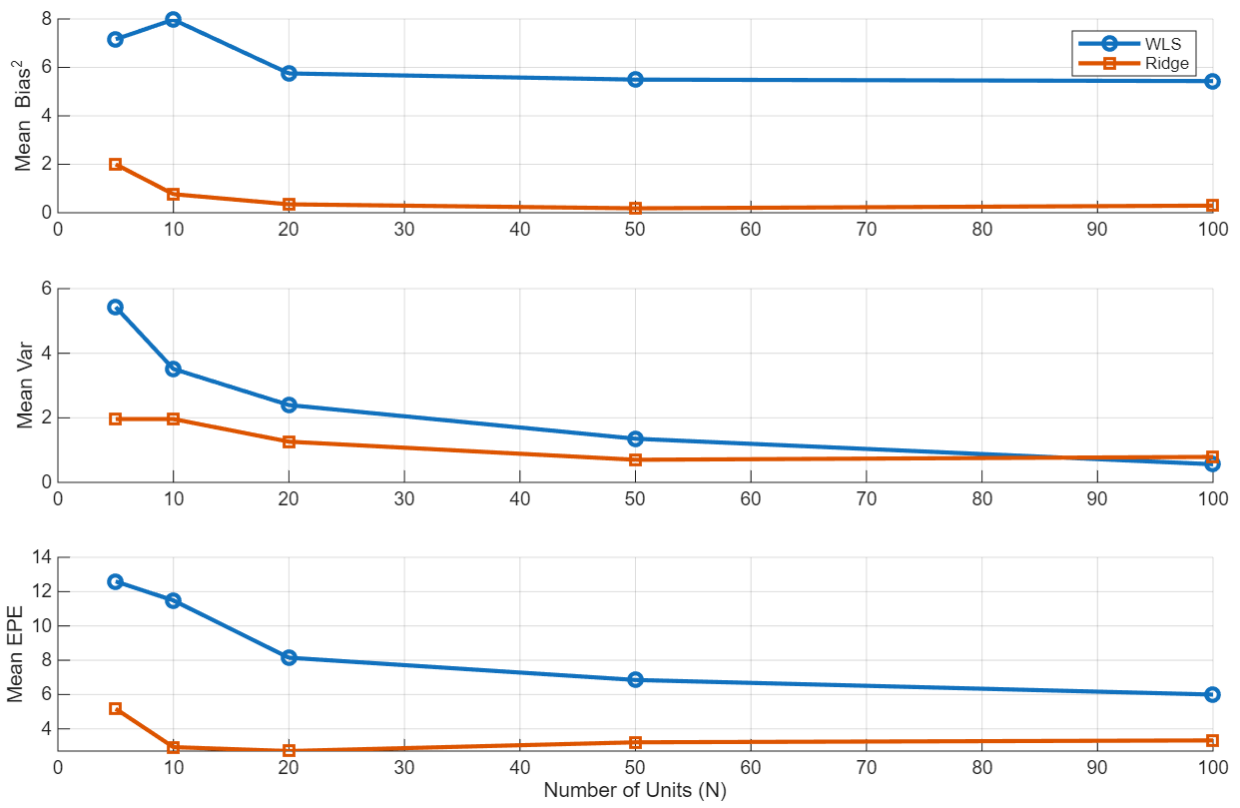
Monte Carlo bias–variance decomposition of emission parameter estimates under WLS and ridge. The mean squared bias, variance, and expected prediction error (EPE) as functions of the number of training units *N* are shown.

We begin by examining the impact of regularization on the accuracy and stability of emission parameter estimation. Figure 2 reports a Monte Carlo bias–variance decomposition of the emission parameter estimates as a function of the training-fleet size *N*, where bias, variance, and expected prediction error (EPE) are averaged over all states and emission parameters.

Across all sample sizes, the ridge-EM estimator exhibits a substantial reduction in estimator variance relative to the unregularized WLS-EM baseline at the cost of a moderate bias for small *N*. This bias–variance trade-off is most pronounced in data-scarce regimes, where WLS-EM suffers from large variability due to poorly conditioned weighted design matrices. As *N* increases, the bias induced by the ridge penalty diminishes, while the variance reduction persists, yielding a uniformly lower EPE for ridge-EM across all configurations.

These results demonstrate that the proposed penalized M-step effectively stabilizes state-wise emission trend estimation under limited effective sample sizes, providing a more reliable parameter foundation for downstream prognostic tasks.

**Figure 3:**
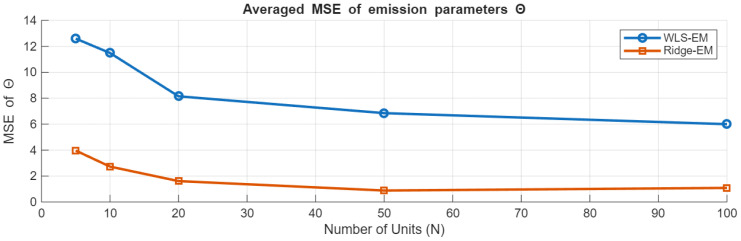
Parameter-space MSE of the emission parameters under WLS-EM and ridge-EM as a function of the number of training units *N*. WLS-EM serves as the unregularized benchmark, while ridge-EM yields consistent reductions in MSE, particularly for small training fleets.

We next consider the accuracy of the full emission parameter vector. Figure 3 reports the parameter-space mean squared error (MSE), defined as the squared $\ell_{2}$ error of the complete emission parameter vector Θ aggregated over all states. Here, WLS-EM corresponds to the unregularized maximum-likelihood benchmark, while ridge-EM introduces bias in exchange for improved numerical conditioning and variance reduction.

Across all sample sizes, ridge-EM achieves lower parameter-space MSE than WLS-EM, with the largest gains observed in small training fleets (N≤20). Notably, the improvement remains non-negligible even at N=100, indicating that ridge regularization remains beneficial beyond extremely data-limited regimes.

**Figure 4:**
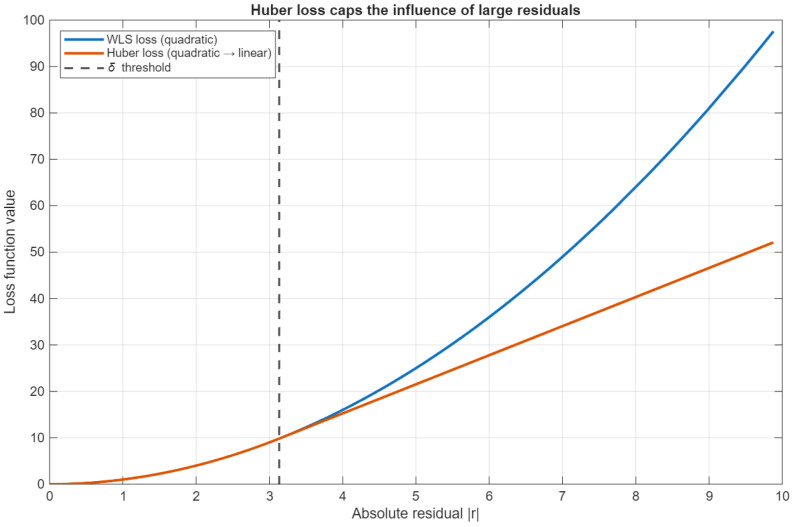
Comparison of quadratic WLS loss and Huber loss as a function of the absolute residual magnitude. The Huber loss coincides with the quadratic loss for small residuals but grows linearly beyond the threshold δ, thereby capping the influence of large deviations in the variance update and improving robustness.

In addition to ridge penalization, we employ robust variance estimation to control the influence of large residuals. Specifically, we adopt a Huber-type loss in the variance update step, which behaves quadratically for small residuals and transitions to linear growth beyond a threshold. This mechanism preserves efficiency under nominal noise while preventing individual large deviations from dominating the variance estimate. Figure 4 illustrates the contrast between the standard quadratic WLS loss and the Huber loss as a function of the absolute residual magnitude.

**Figure 5:**
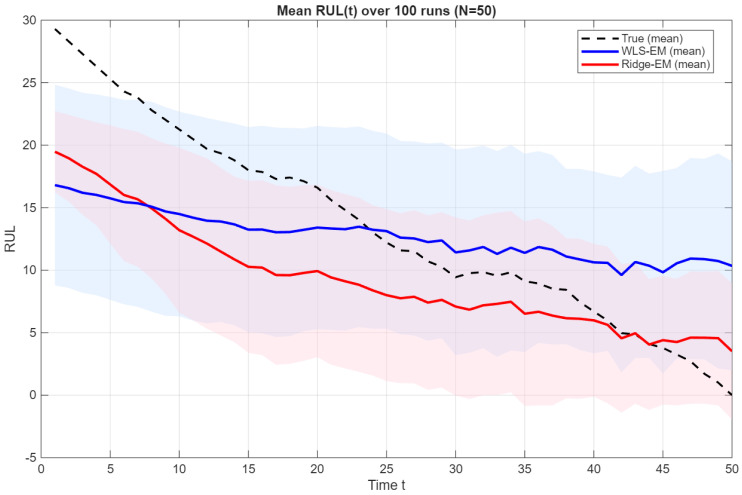
Mean RUL trajectories over 100 simulations for N=50. The dashed line denotes the true RUL; solid lines show the mean predictions for WLS-EM and ridge-EM, with shaded bands indicating empirical variability.

We next evaluate prognostic performance using the weighted RUL estimator introduced in Section 2.4. Figure 5 reports the mean RUL trajectories for N=50 across Monte Carlo runs. The unregularized WLS-EM exhibits a systematic tendency to overestimate the remaining life, most prominently in the mid-life region of the degradation process, where prediction uncertainty is highest. This behavior reflects a positive bias in the average RUL estimates, arising from instability in the emission parameter estimates and the resulting latent-state posteriors. By contrast, ridge-EM yields mean trajectories that remain closer to the true RUL curve and produces noticeably narrower variability bands, indicating both improved stability and reduced dispersion in the predictions.

Table 3 summarizes RUL prediction accuracy across all training fleet sizes using the root mean squared error (RMSE) and the predicted and true RUL trajectories averaged over Monte Carlo replications. Ridge-EM improves prediction accuracy by approximately 2–3 units for all values of *N*, with the largest relative gains at moderate fleet sizes.

**Figure 6:**
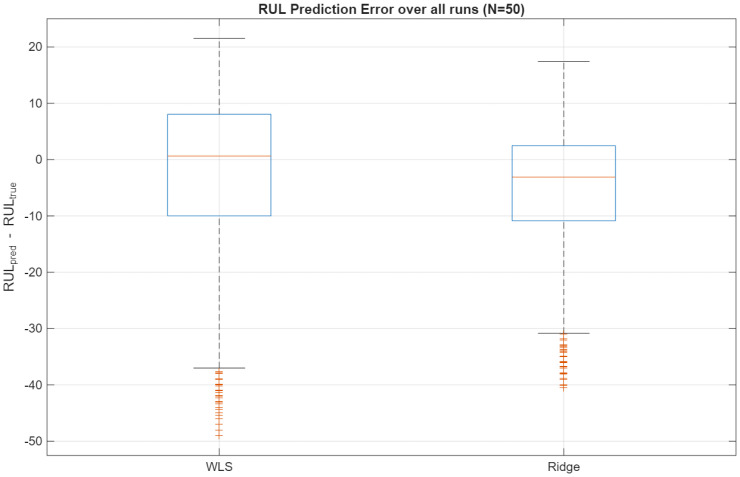
Distribution of RUL prediction errors over all Monte Carlo runs for N=50. Ridge-EM reduces the spread of errors and suppresses extreme negative outliers compared with WLS-EM.

To better understand this improvement, Figure 6 presents the distribution of RUL prediction errors at a representative time point for N=50. The WLS-EM baseline yields a wider error distribution with several large negative outliers (severe underestimation of remaining life), whereas ridge-EM produces a more concentrated and symmetric error profile, indicating improved robustness against local anomalies in the simulated trajectories. Beyond visual comparison, a paired Monte Carlo analysis demonstrates that the reduction in RUL prediction error achieved by ridge-EM at N=50 is statistically significant, with a 95% confidence interval for the RMSE difference that does not include zero.

Finally, we examine the bias–variance decomposition of the RUL mean squared error. Across all simulation settings, WLS-EM exhibits relatively small bias but substantially larger variance, particularly for small and moderate training fleet sizes. This variance inflation dominates the overall error, leading to higher RUL MSE. In contrast, ridge-EM yields a pronounced reduction in variance while also stabilizing the bias component, resulting in a lower overall expected prediction error (EPE).

This behavior is summarized quantitatively in Table 4 for the representative case N=50, where ridge-EM achieves a substantial reduction in both variance and total MSE.

Overall, RUL errors under WLS-EM are dominated by estimator variance, whereas ridge-EM achieves lower total error by substantially stabilizing RUL predictions at the cost of a small bias increase.

## 4. Real-World Case Study: NASA C-MAPSS

To demonstrate the practical applicability of the proposed ridge-regularized robust HMM framework, we evaluate it on the widely used NASA C-MAPSS turbofan degradation dataset (FD001). This dataset is commonly employed in prognostics benchmarks due to its single-operating-condition structure and monotone degradation behavior, making it well-suited for simple-failure latent-state modeling.

### 4.1. Dataset and Preprocessing

FD001 contains multivariate run-to-failure trajectories from 100 training and 100 test engines. Each record includes the unit identifier, cycle index, three operating condition variables, and 21 sensor channels. In this study, we use all 21 sensor channels to construct a univariate health indicator (HI). The operating condition variables are excluded from the analysis as FD001 corresponds to a single operating regime and these variables do not provide additional discriminative information.

Sensor channels are standardized using z-score normalization based on the training-fleet statistics. Specifically, for each sensor channel, the training mean and standard deviation are computed and the same transformation is applied to both training and test units.

A univariate HI is then constructed via principal component analysis (PCA) trained on the normalized training sensor matrix. The first principal component is retained, and its sign is oriented using its correlation with the cycle index so that the HI reflects a consistent direction degradation over time. PCA is employed here as a widely adopted health indicator construction approach for the FD001 dataset, widely used in prior studies to capture dominant degradation trends, while the focus of this work remains on estimation robustness rather than an optimized feature extraction strategy. For the test units, the HI is obtained by applying the learned loading vector to the normalized sensor matrix. The resulting HI trajectories exhibit the expected slow decline during healthy operation, followed by a sharp descent near failure.

Each unit is modeled using a *K*-state simple-failure HMM, where latent states 1,…,K−1 represent progressive degradation levels and state *K* is absorbing. For the real-data analysis, the number of latent states is selected in a data-driven manner using the Bayesian information criterion (BIC), which is widely adopted for model order selection in HMM-based prognostics. To compare competing models with different values of *K*, the BIC is computed from the unpenalized log-likelihood, consistent with standard practice for penalized likelihood models.

For the simple-failure HMM considered here, the total number of free parameters is

d=(K−1)+3K,

corresponding to K−1 free transition probabilities under the absorbing-state structure and three emission parameters per state. The BIC is therefore defined as

$$\mathrm{BIC}=-2\;\ell \left(\hat{\Theta }\right)\;+\;d\;\log\;\left(T_{\mathrm{tot}}\right),\;$$

where $T_{\mathrm{tot}}=\sum_{i=1}^{N}T_{i}$ denotes the total number of observed time points across all units. This formulation is consistent with prior HMM-based condition monitoring and prognostic studies [27,28,29]. Based on this criterion, K=6 achieves the lowest BIC among the candidate models and is therefore adopted for all subsequent real-data experiments. The corresponding log-likelihood and BIC values for K∈{4,5,6} are summarized in Table 5.

### 4.2. Model Training and State Progression

Model parameters are learned using both WLS-EM and ridge-EM from a structured and deterministic initialization. The initial state distribution $\pi_{0}$ is concentrated on the healthiest state, and the transition probability matrix is initialized with a monotone stay-forward structure and an absorbing failure state. Emission parameters are initialized from a global linear regression fit to the available data; small deterministic intercept offsets are introduced across states to avoid identical initial emissions, while the slope and noise parameters are kept common across states. Ridge regularization is applied only to the slope parameters, with penalty strength $\lambda_{k}$ selected via the weighted 1 − SE cross-validation rule described in Section 3.2 (see Appendix C).

**Figure 7:**
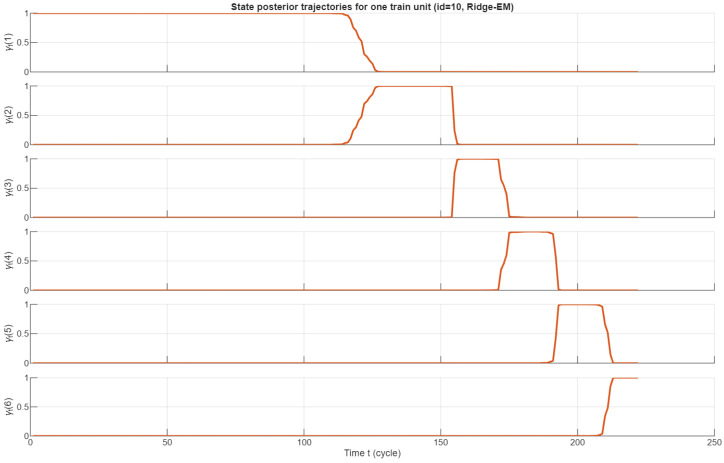
Posterior-state probabilities for a representative FD001 unit. The monotone progression across hidden states is consistent with the simple-failure assumption. Ridge regularization produces stable parameter estimates without altering latent-state segmentation.

Figure 7 shows posterior-state probabilities $\gamma_{t}\left(k\right)$ for a representative training unit. The unit progresses monotonically through the latent degradation levels, transitioning approximately at t∈{120,145,170,195,210} before reaching the absorbing failure state. Importantly, WLS-EM and ridge-EM produce nearly identical state segmentation, demonstrating that ridge penalization stabilizes parameter estimation without altering the underlying physical degradation structure. However, ridge-EM yields smoother and less noisy $\gamma_{t}\left(k\right)$ trajectories, especially within late-stage states where the WLS solution exhibits small but noticeable fluctuations. This improved stability directly influences RUL estimation as RUL predictions depend on the posterior distribution over the transient degradation states.

### 4.3. RUL Prediction Results

We evaluate the RUL prediction performance of the proposed methods on the FD001 test set using standard accuracy and reliability metrics. Table 6 summarizes the test RMSE and MAE, together with empirical coverage rates for error tolerance δ∈{5,10,20} periods. Let $e_{j}={\hat{\mathrm{RUL}}}_{j}-{\mathrm{RUL}}_{j}$ denote the prediction error for test unit *j*. The coverage rate at tolerance δ is defined as the fraction of test units whose absolute prediction error satisfies $|e_{j}|\leq \delta$.

Across all metrics, ridge-EM consistently outperforms the unregularized WLS-EM baseline. In particular, ridge-EM achieves substantially lower RMSE and MAE while also exhibiting higher empirical coverage at all error thresholds, indicating improved accuracy and calibration of the RUL predictions.

**Figure 8:**
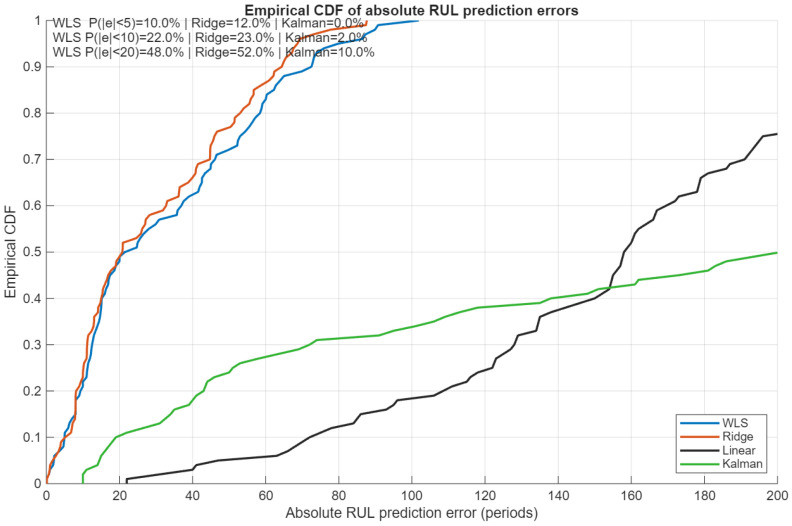
Empirical CDF of absolute RUL prediction errors on the FD001 test set. Ridge-EM consistently attains higher coverage at all error levels compared to WLS-EM and the considered benchmark methods.

Figure 8 complements these aggregate metrics by showing the empirical cumulative distribution function (CDF) of the absolute RUL prediction errors. In addition to WLS-EM and the proposed ridge-EM, the figure includes two fundamental benchmark models: a linear degradation regression and a simple Kalman filter-based state-space estimator. These baselines represent classical trend-based and state-space RUL estimation approaches that are widely adopted in industrial predictive maintenance workflows (e.g., MathWorks Predictive Maintenance Toolbox [30]). Across the full error range, the ridge-EM curve dominates that of WLS-EM, indicating a uniformly higher probability of achieving smaller prediction errors.

**Figure 9:**
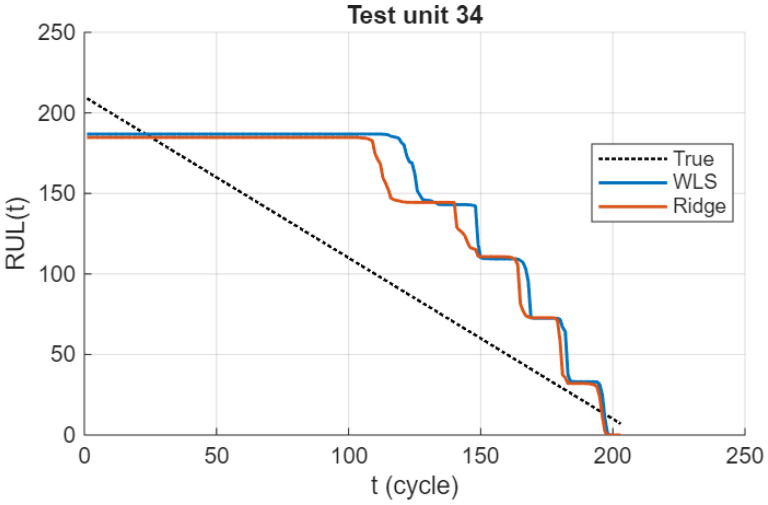
RUL trajectories for a representative FD001 test unit. Ridge-EM stabilizes predictions near state transitions while preserving global accuracy.

Figure 9 shows the RUL trajectories for a representative FD001 test unit. Both WLS-EM and ridge-EM capture the overall degradation trend and the countdown toward failure. Differences are most pronounced around inferred state transitions, where WLS-EM exhibits abrupt changes in the predicted RUL. In contrast, ridge-EM yields less variable transitions, reflecting increased stability of the underlying emission parameter estimates. This behavior is consistent with the variance-reducing effect of ridge regularization under state-wise data sparsity rather than a systematic shift in bias.

**Figure 10:**
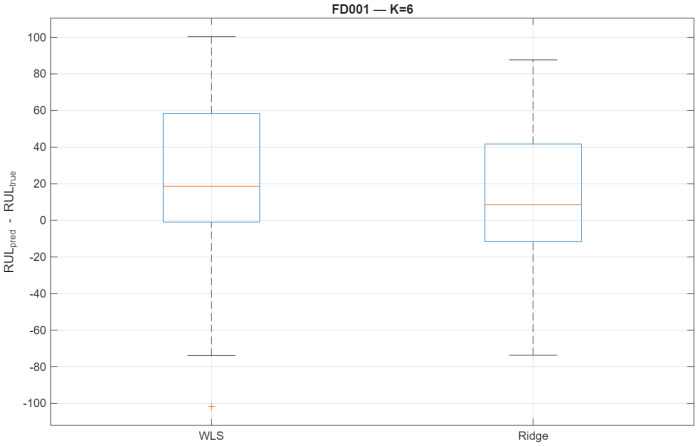
Distribution of RUL prediction errors for FD001. Ridge-EM reduces the frequency and severity of large negative prediction errors.

Finally, Figure 10 summarizes the distribution of prediction errors across all test engines, showing a tighter and more symmetric error profile for ridge-EM.

Overall, the results demonstrate that the proposed ridge-regularized EM framework provides a more stable and reliable basis for RUL prediction under limited and noisy run-to-failure data. Performance is evaluated using complementary metrics, including RMSE, MAE, error-coverage probabilities, and bias–variance decomposition, which jointly assess predictive accuracy, robustness, and estimator stability. Across both simulation studies and real-data experiments, the proposed approach consistently improves prediction reliability relative to the unregularized WLS-EM baseline while preserving analytical tractability and model transparency.

## 5. Results

This section presents the empirical evaluation of the proposed methodology based on both Monte Carlo simulation studies and the NASA C-MAPSS FD001 benchmark dataset. The simulation experiments focus on parameter estimation accuracy, cross-validation behavior, EM convergence properties, and robustness under varying noise and fleet-size conditions, with detailed results reported in Section 3.3. Complementarily, the real-data analysis investigates posterior-state evolution, unit-specific remaining useful life (RUL) trajectories, and aggregate prediction performance, as discussed in Section 4.3.

**Figure 11:**
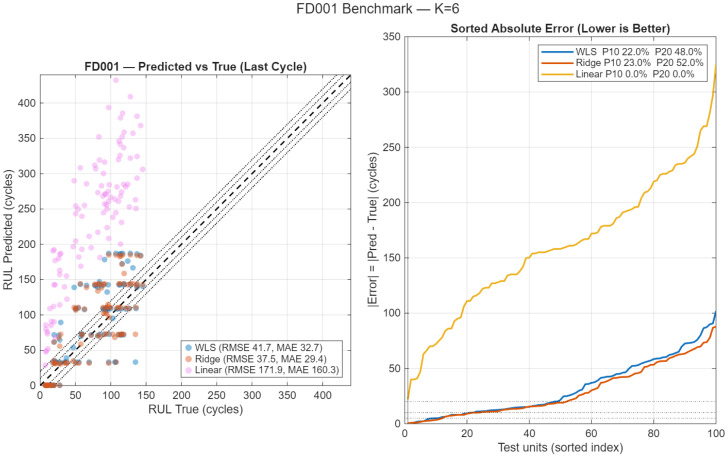
Benchmark comparison on the NASA C-MAPSS FD001 dataset. (**Left**): Predicted versus true RUL at the last cycle for WLS-EM, ridge-EM, and linear regression, including the identity line and tolerance bands. (**Right**): Sorted absolute RUL prediction errors, highlighting the relative error distributions of the competing methods. Ridge-EM exhibits reduced dispersion and improved accuracy compared with WLS-EM and the linear baseline.

Figure 11 compares the RUL prediction performance of the proposed ridge-EM approach against the WLS-EM baseline and a linear regression benchmark on the FD001 dataset, evaluated at the last available cycle of each test unit. The left panel shows predicted versus true RUL values, together with the identity line and tolerance bands, while the right panel reports sorted absolute prediction errors, facilitating a direct comparison of error distributions across methods.

The results indicate that ridge-EM achieves a tighter concentration around the identity line and substantially reduced dispersion relative to the WLS-EM estimator. In contrast, the linear regression benchmark exhibits pronounced bias and large prediction variance, particularly for medium and long RUL horizons. The sorted absolute error curves further confirm that ridge-EM consistently dominates the competing methods across the full range of test units, yielding higher proportions of predictions within practical tolerance thresholds.

Overall, these findings demonstrate that the proposed ridge-EM estimator provides (i) improved numerical stability during EM iterations, (ii) reduced variance in emission-parameter estimates, and (iii) more reliable short- and medium-horizon RUL predictions compared with WLS-EM. Importantly, these advantages persist across different fleet sizes, noise levels, and real-world sensor trajectories, supporting the robustness and practical relevance of the proposed approach.

## 6. Discussion

The real-data analysis and the Monte Carlo experiments together provide a coherent picture of how ridge-regularized and Huber-robust updates improve the statistical behavior of HMM-based prognostic models.

First, ridge penalization stabilizes the estimation of state-dependent slopes and variances, reducing the sensitivity of the EM updates to early- or late-stage data scarcity. This manifests most clearly in the posterior-state trajectories $\gamma_{t}\left(k\right)$, which become smoother and less noisy without altering the underlying degradation segmentation. Because the weighted RUL estimator is a linear functional of $\gamma_{t}$, this variance reduction directly translates into improved RUL stability.

Second, both the simulation and the FD001 analysis show that the regularized estimator attains similar or slightly improved mean accuracy while substantially reducing severe underestimation errors. From a bias–variance perspective, the Monte Carlo experiments confirm that ridge-EM reduces variance markedly while introducing only negligible bias, leading to consistently lower expected prediction error (EPE) across all training-fleet sizes. This property is particularly important in safety-critical prognostics, where controlling the variability of short-horizon predictions is often more crucial than marginal reductions in mean error. In particular, while conservative RUL underestimation may be preferred to avoid overly optimistic decisions, high-variance estimators can produce erratic and unpredictable predictions, including extreme errors driven by local anomalies. By suppressing such variability and extreme outliers, the proposed approach yields more reliable RUL estimates while remaining compatible with conservative maintenance decision rules.

Third, the proposed algorithm enhances numerical robustness while preserving the standard computational structure of the classical HMM-EM framework. The ridge-regularized M-step modifies the closed-form emission updates in a principled manner, improving the conditioning of the estimation problem without introducing additional iterative layers or auxiliary optimization routines. Importantly, ridge penalization alone alleviates ill-conditioning due to limited effective sample sizes but remains sensitive to outliers, while robust variance estimation mitigates outlier influence without resolving instability in slope estimation under state-dependent data scarcity. By integrating state-dependent slope penalization with robust variance estimation inside the EM loop, the proposed framework jointly stabilizes parameter estimation and latent-state inference. As a result, the method improves estimator stability and reliability while retaining the practical simplicity and interpretability of conventional HMM-based approaches.

Building on these considerations, a related modeling choice concerns the use of linear state-dependent emission models. In this work, linear emissions were deliberately adopted to preserve parameter identifiability, numerical stability, and analytical tractability within the EM framework, particularly under limited run-to-failure data. While real degradation processes may exhibit nonlinear trends, such behavior can often be approximated through transitions among multiple latent health states, yielding an effective piecewise-linear representation. Alternatively, the proposed framework can be extended to incorporate nonlinear emission functions, such as polynomial or basis-expansion models, within each state, as well as alternative health indicator constructions, provided that estimation stability and interpretability are preserved. Investigating these extensions and comparative evaluations across different feature representations and their impact on estimation stability and robustness constitutes an important direction for future research.

Similarly, although ridge regularization improves estimator stability, more flexible penalty structures, such as adaptive or hierarchical formulations, may offer additional gains in highly heterogeneous fleets. The FD001 dataset serves as a benchmark in this study, and extending the evaluation to other real-world systems would help to further assess the generalization of the proposed framework.

In summary, the proposed ridge-regularized and Huber-robust HMM provides a principled modeling framework for improving parameter stability, latent-state smoothness, and short-horizon RUL reliability while remaining fully compatible with standard HMM-based prognostic pipelines. By jointly addressing slope ill-conditioning and variance inflation within the EM procedure, the proposed approach offers a robust and practically applicable enhancement to classical HMM-based degradation modeling.

## 7. Conclusions

This work presented a statistically principled extension of HMM-based hard-failure prognostics by introducing ridge-regularized emission estimation and a Huber-type robust variance update within the EM framework. Rather than modifying the latent-state structure or inference mechanism, the proposed approach directly targets a key but often overlooked source of instability in HMM-based RUL prediction: the high variance and poor numerical conditioning of posterior-weighted state-wise regression updates under limited run-to-failure data.

Through comprehensive Monte Carlo experiments and a real-world evaluation on the NASA C–MAPSS FD001 dataset, the ridge-regularized EM formulation was shown to substantially reduce estimator variance while introducing negligible bias. These improvements translate into smoother latent-state posterior trajectories and markedly more stable short-horizon RUL predictions. In contrast, the conventional WLS-EM approach remains sensitive to data scarcity and local measurement irregularities, which can lead to erratic parameter updates and unreliable remaining-life estimates.

By preserving the probabilistic structure, analytical RUL formulation, and model transparency of classical HMM-based joint models, the proposed method offers a minimal yet effective alternative to more complex or black-box prognostic approaches, particularly in small fleets and safety-critical settings. From a practical perspective, the improved stability of short-horizon RUL predictions directly supports more reliable maintenance scheduling and reduces the risk of overly conservative or delayed interventions under limited data availability. Future research may incorporate nonlinear emission structures, covariate-dependent transition dynamics, or online (recursive) extensions to further enhance applicability in real-time PHM settings and heterogeneous fleets.

## Figures and Tables

**Table 1:** True transition probability matrix used in the simulation study.

	State 1	State 2	State 3	State 4 (Absorbing)
State 1	0.9	0.1	0	0
State 2	0	0.9	0.1	0
State 3	0	0	0.9	0.1
State 4	0	0	0	1

**Table 2:** True emission parameters $\left(\eta_{0,k},\eta_{1,k},\sigma_{k}\right)$ for each latent state.

State *k*	$\eta_{0,k}$	$\eta_{1,k}$	$\sigma_{k}$
1	1.0	0.1	0.5
2	2.0	0.2	0.5
3	3.0	0.3	0.5
4	4.0	0.4	0.5

**Table 3:** Average RUL RMSE over 100 Monte Carlo runs.

*N*	RMSE (WLS)	RMSE (Ridge)	Improvement (Ridge–WLS)
5	11.18	9.58	−1.60
10	11.67	9.58	−2.09
20	12.81	9.27	−3.54
50	10.96	8.61	−2.35
100	11.25	9.14	−2.11

**Table 4:** Bias–variance decomposition of RUL prediction error for N=50. The time-averaged squared bias, variance, and mean squared error (MSE) over all Monte Carlo runs are shown.

Method	Bias^2^	Variance	MSE
WLS-EM	28.90	63.80	143.30
Ridge-EM	20.60	44.45	96.40

**Table 5:** Model order selection for the FD001 dataset using BIC.

Number of States (*K*)	Log-Likelihood	BIC
4	−26,717.9	53,584.8
5	−24,840.4	49,869.5
6	−23,508.4	47,245.4

**Table 6:** RUL prediction performance on the FD001 test set. The root mean squared error (RMSE), mean absolute error (MAE), and empirical coverage rates are reported, defined as the fraction of test units whose absolute prediction error does not exceed the specified tolerance.

Method	RMSE	MAE	|e|≤5	|e|≤10	|e|≤20
WLS-EM	50.54	40.33	10.0%	22.0%	48.0%
Ridge-EM	37.51	29.37	12.0%	23.0%	52.0%

**Figure A1:**
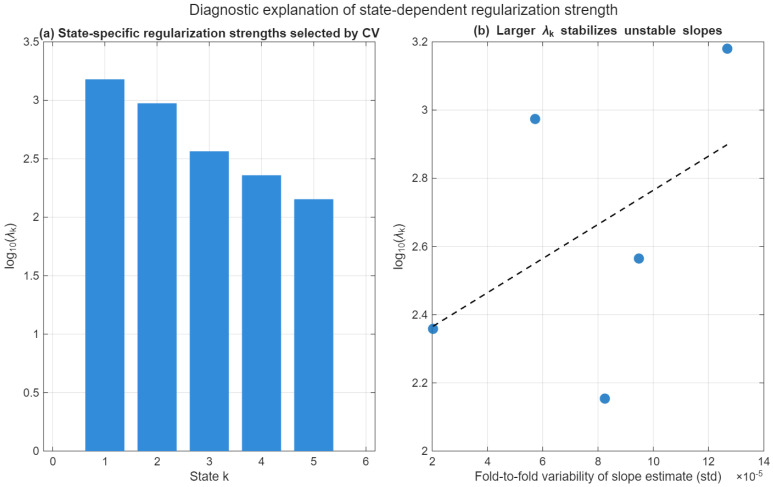
Diagnostic explanation of state-dependent regularization strength in the real-world case study. (**a**) State-specific regularization strengths $\lambda_{k}$ selected via the weighted 1 − SE cross-validation rule. (**b**) Relationship between the selected $\lambda_{k}$ values and the cross-validation variability of the corresponding slope estimates. The dashed line is shown as a visual guide.

## Data Availability

The NASA C–MAPSS FD001 turbofan engine degradation dataset used in this study is publicly available through the Prognostics Center of Excellence (PCoE) at NASA Ames Research Center: https://www.nasa.gov/intelligent-systems-division/discovery-and-systems-health/pcoe/pcoe-data-set-repository/ (accessed on 13 February 2026). All simulation codes, parameter settings, and scripts used for generating the results of the Monte Carlo experiments are available from the corresponding author upon reasonable request. No proprietary or confidential data were used in this study.

## Abbreviations

The following abbreviations are used in this manuscript:

HMM	Hidden Markov model
EM	Expectation–maximization
WLS	Weighted least squares
RUL	Remaining useful life
TPM	Transition probability matrix
CM	Condition monitoring
HI	Health indicator
MAD	Median absolute deviation
BIC	Bayesian information criterion
CV	Cross-validation
RMSE	Root mean squared error
MAE	Mean absolute error
EPE	Expected prediction error
NASA C–MAPSS	NASA Commercial Modular Aero-Propulsion System Simulation dataset

## Appendix A. Closed-Form WLS Emission Update

For reference, we summarize the closed-form weighted least squares (WLS) estimators used as the unregularized baseline in the EM algorithm.

For each transient state k∈{1,…,K−1}, define the weighted sample means

(A1) $${\bar{y}}_{k}=\frac{\sum_{i,t}\gamma_{it}\left(k\right)\;y_{it}}{\sum_{i,t}\gamma_{it}\left(k\right)},\;{\bar{t}}_{k}=\frac{\sum_{i,t}\gamma_{it}\left(k\right)\;t}{\sum_{i,t}\gamma_{it}\left(k\right)}.$$

The WLS estimators of the slope and intercept are given by

(A2) $${\hat{\eta }}_{1k}=\frac{\sum_{i,t}\gamma_{it}\left(k\right)\;\left(t-{\bar{t}}_{k}\right)\left(y_{it}-{\bar{y}}_{k}\right)}{\sum_{i,t}\gamma_{it}\left(k\right)\;{\left(t-{\bar{t}}_{k}\right)}^{2}},$$

(A3) $${\hat{\eta }}_{0k}={\bar{y}}_{k}-{\hat{\eta }}_{1k}\;{\bar{t}}_{k}.$$

Given the fitted values

$${\hat{y}}_{it,k}={\hat{\eta }}_{0k}+{\hat{\eta }}_{1k}t,$$

the state-specific noise variance is estimated as

(A4) $${\hat{\sigma }}_{k}^{2}=\frac{\sum_{i,t}\gamma_{it}\left(k\right)\;{\left(y_{it}-{\hat{y}}_{it,k}\right)}^{2}}{\sum_{i,t}\gamma_{it}\left(k\right)}.$$

These expressions correspond to the standard WLS solution obtained from the state-wise maximization of the expected complete-data log-likelihood under Gaussian emissions.

## Appendix B. Bias–Variance Decomposition for the RUL Estimator

This appendix provides the theoretical background underlying the bias-variance analysis of the remaining useful life (RUL) estimator, as illustrated empirically in Section 3.3. The goal is to clarify how ridge regularization reduces the variance of RUL predictions while introducing only minimal bias.

Let ${\mathrm{RUL}}_{i}\left(t^{*}\right)=\tau_{i}-t^{*}$ denote the true (latent) remaining useful life of unit *i* at time $t^{*}$, and let ${\hat{\mathrm{RUL}}}_{i}\left(t^{*}\right)$ denote its estimator defined in (34). The mean squared prediction error admits the decomposition

(A5) $$E\;\left[{\left({\hat{\mathrm{RUL}}}_{i}\left(t^{*}\right)-{\mathrm{RUL}}_{i}\left(t^{*}\right)\right)}^{2}\right]={\left(E\left[{\hat{\mathrm{RUL}}}_{i}\left(t^{*}\right)\right]-{\mathrm{RUL}}_{i}\left(t^{*}\right)\right)}^{2}+\mathrm{Var}\;\left({\hat{\mathrm{RUL}}}_{i}\left(t^{*}\right)\right).$$

In the proposed HMM framework, the RUL estimator is given by the weighted hitting-time expression introduced in Section 2.4:

(A6) $$\hat{\mathrm{RUL}}\left(t\right)=\gamma_{t}^{⊤}F1,$$

where $\gamma_{t}$ is the smoothed posterior-state vector and $F={\left(I-Q\right)}^{-1}$ is the fundamental matrix of the transient-state transition submatrix *Q*. The vector F1 contains the expected numbers of future steps until absorption starting from each latent degradation state.

Because the estimator in (A6) depends linearly on both the posterior probabilities $\gamma_{t}$ and the emission parameters $\left(\eta_{0k},\eta_{1k},\sigma_{k}^{2}\right)$, variance in either component propagates to the RUL prediction. Ridge regularization reduces the dispersion of these emission-parameter estimates, which in turn stabilizes $\gamma_{t}$ and yields lower overall prediction variance. This theoretical mechanism is consistent with the empirical results reported in Section 3.3.

## Appendix C. Diagnostic Analysis of State-Dependent Regularization

This appendix provides additional diagnostic results to aid the interpretation of the state-dependent regularization strengths selected in the real-world case study. As described in the main text, the regularization parameters $\lambda_{k}$ are selected solely based on validation performance using the weighted 1 − SE cross-validation rule. The analyses presented here are post hoc diagnostics and do not influence model training or hyperparameter selection. Their purpose is to provide intuition for why certain states benefit from stronger regularization than others.

Figure A1 illustrates how the state-dependent regularization strengths selected by cross-validation vary across degradation states in the real-world case study. Panel (a) shows that earlier transient states are assigned stronger regularization, while later states require progressively weaker penalties. Panel (b) relates the selected $\lambda_{k}$ values to the stability of the corresponding slope estimates across cross-validation splits, indicating that states with less stable slope estimates tend to be associated with larger regularization strengths. This suggests that stronger regularization helps to stabilize emission parameter estimation in states that are more difficult to estimate reliably.

These diagnostic results complement the main analysis by providing additional insight into the behavior of state-dependent regularization in the real-world case study.
